# SARS-CoV-2 pandemic and research gaps: Understanding SARS-CoV-2 interaction with the ACE2 receptor and implications for therapy

**DOI:** 10.7150/thno.48076

**Published:** 2020-06-12

**Authors:** Prasun K. Datta, Fengming Liu, Tracy Fischer, Jay Rappaport, Xuebin Qin

**Affiliations:** 1 Division of Comparative Pathology, Tulane National Primate Research Center, Covington, LA 70433, USA.; 2 Department of Immunology and Microbiology, Tulane University School of Medicine, New Orleans, LA 70112, USA.

**Keywords:** COVID-19, ACE2, spike protein, pathogenesis, and animal model

## Abstract

The COVID-19 pandemic is an emerging threat to global public health. While our current understanding of COVID-19 pathogenesis is limited, a better understanding will help us develop efficacious treatment and prevention strategies for COVID-19. One potential therapeutic target is angiotensin converting enzyme 2 (ACE2). ACE2 primarily catalyzes the conversion of angiotensin I (Ang I) to a nonapeptide angiotensin or the conversion of angiotensin II (Ang II) to angiotensin 1-7 (Ang 1-7) and has direct effects on cardiac function and multiple organs via counter-regulation of the renin-angiotensin system (RAS). Significant to COVID-19, ACE2 is postulated to serve as a major entry receptor for SARS-CoV-2 in human cells, as it does for SARS-CoV. Many infected individuals develop COVID-19 with fever, cough, and shortness of breath that can progress to pneumonia. Disease progression promotes the activation of immune cells, platelets, and coagulation pathways that can lead to multiple organ failure and death. ACE2 is expressed by epithelial cells of the lungs at high level, a major target of the disease, as seen in post-mortem lung tissue of patients who died with COVID-19, which reveals diffuse alveolar damage with cellular fibromyxoid exudates bilaterally. Comparatively, ACE2 is expressed at low level by vascular endothelial cells of the heart and kidney but may also be targeted by the virus in severe COVID-19 cases. Interestingly, SARS-CoV-2 infection downregulates ACE2 expression, which may also play a critical pathogenic role in COVID-19. Importantly, targeting ACE2/Ang 1-7 axis and blocking ACE2 interaction with the S protein of SARS-CoV-2 to curtail SARS-CoV-2 infection are becoming very attractive therapeutics potential for treatment and prevention of COVID-19. Here, we will discuss the following subtopics: 1) ACE2 as a receptor of SARS-CoV-2; 2) clinical and pathological features of COVID-19; 3) role of ACE2 in the infection and pathogenesis of SARS; 4) potential pathogenic role of ACE2 in COVID-19; 5) animal models for pathological studies and therapeutics; and 6) therapeutics development for COVID-19.

## Introduction

A recently identified novel betacoronavirus, severe acute respiratory syndrome coronavirus-2 (SARS-CoV-2), is the etiological agent of coronavirus disease-2019 (COVID-19). COVID-19 emerged in December 2019 in Wuhan City, China and rapidly spread to the rest of world. With a 4-12% mortality rate, COVID-19 is an emerging threat to global public health. According to the World Health Organization (WHO), China reported a cluster of pneumonia cases in Wuhan, Hubei province on December 31, 2019. It was reported that no deaths were associated with this pneumonia outbreak, which involved 44 patients as of January 4, 2020. On January 5, 2020, WHO announced that the pneumonia outbreak was due to a new virus that originated in the Huanan Seafood market in Wuhan, China, on January 12, 2020, the virus was confirmed as a novel coronavirus (nCoV). The first case of nCoV outside of China was reported in Thailand on January 13, 2020.

On the following day, the WHO announced that there was limited human-to-human transmission of nCoV, mainly through family members, and suggested the possibility of a wider outbreak. On January 30, 2020, the WHO declared that 2019-nCoV was a Public Health Emergency of International Concern (PHEIC). On February 11, 2020, 2019‐nCoV was named severe acute respiratory syndrome coronavirus 2 (SARS‐CoV‐2) by the coronavirus study group of the International Committee on Taxonomy of Viruses and the WHO officially named the disease caused by the 2019‐nCoV as coronavirus disease (COVID‐19). A month later, March 11, 2020, the WHO declared COVID-19 a pandemic, and SARS-CoV-2 infection has since reached pandemic status worldwide. The worldwide spread of this virus from China is associated with international travel and subsequent communal transmission.

Currently, the virus is aggressively and rapidly spreading, infecting millions of people globally, and causing high mortality and morbidity. Indeed, compared to SARS-CoV and MERS-CoV, SARS-CoV-2 is more virulent, infecting a greater number of individuals world-wide than these earlier viruses. The available public health tools to control transmission are passive approaches, such as isolation and quarantine, social distancing, and community containment measures. Moreover, after the current crisis abates, there remains a high probability of a reemergence of SRAS-CoV-2 and COVID-19 in the fall or upcoming years, as there are currently no vaccines or specific treatment available for COVID-19.

**Figure 1:**
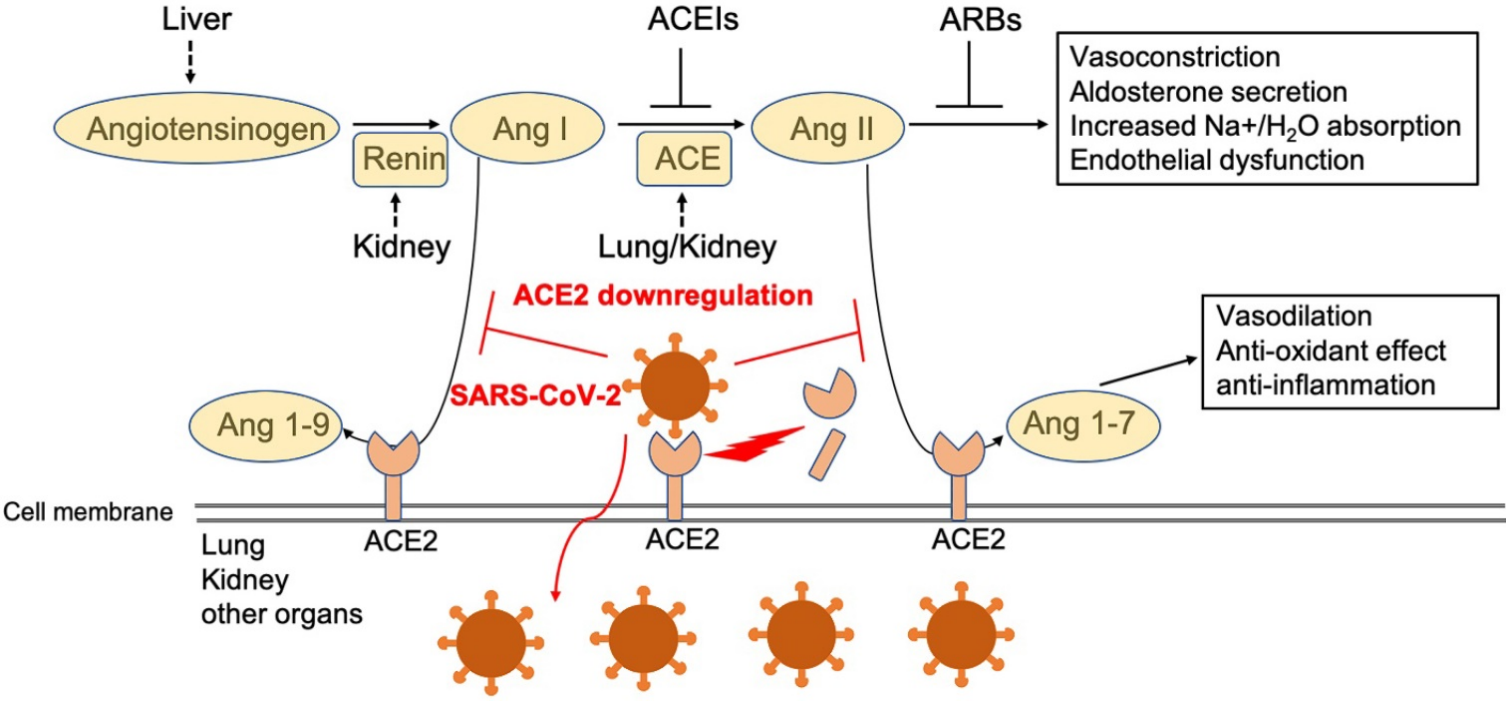
** Function of ACE2 in renin-angiotensin system (RAS) and SARS-CoV2 downregulation of ACE2 level in membrane.** Angiotensinogen is converted by renin to angiotensin I (Ang I). Ang I is subsequently converted to angiotensin II (Ang II) by ACE, which is expressed on the surface of endothelial cells in lung and kidney. Angiotensin-converting enzyme inhibitors (ACEIs) inhibit the production of Ang II and angiotensin receptor blockers (ARBs) inhibits the binding of Ang II to angiotensin receptors. ACE2 negatively regulates the function of ACE by converting Ang I to Ang 1-9 and Ang II to Ang 1-7. SARS-CoV-2 interacts with ACE2 and infects ACE2-expressing epithelial and endothelial cells in lung and other organs, leading to the down-regulation of ACE2 on endothelium of lung and presumably, other organs, such as kidney. The downregulation of ACE2 leads to unopposed Ang II accumulation, which may accelerate the progress of COVID-19 via increased activity of RAS.

While previous recognition of SARS pathogenesis greatly facilitates our understanding of the pathogenesis of COVID-19, our current understanding remains limited and is based largely on clinical observations and a few autopsy findings. A better understanding of the pathogenesis will help us develop better, more specific treatment and prevention strategies for COVID-19. One potential target for therapeutic intervention is angiotensin converting enzyme 2 (ACE2), a peptidase in the renin-angiotensin system (RAS) (Figure 1). ACE2 plays a crucial role in SARS infection and is believed to serve as a major entry receptor for SARS-CoV-2 in humans (Figure 1).

In infected patients, SARS-CoV-2 mainly infects the type II pneumocytes in lungs, and but also infects proximal tubule epithelial cells in kidney in severe cases 1. These cells express its receptor ACE2, which facilitates viral entry 2, 3. The endothelial cells in a variety of tissues may be also infected by the virus, although they appear to express little to no ACE2. Infection leads to the downregulation of ACE2 (Figure 1), which impacts the function of Ang II and RAS in a variety of tissues, including lung, heart, vasculature, and kidney, and may facilitate the progression of COVID-19 from mild and moderate to more severe disease (Figure 1). Indeed, direct infection of endothelial cells may explain some of the clinical features of advanced disease, including massive activation of coagulation pathways and platelets, as well as immune cells, such as monocytes and neutrophils in the lung, heart, and kidney. These observations suggest that ACE2 may play a critical pathogenic role in COVID-19, which warrants further extensive investigation. Here, we review the current view of ACE2 in the pathogenesis of SARS-CoV-2 infection.

## ACE2 serves as a receptor for SARS-CoV-2

### ACE2 as a receptor for SARS-CoV and SARS-CoV-2

ACE2 serves as an entry receptor for SARS-CoV-2 and SARS-CoV in humans by binding to the viral envelope protein spike (S) protein, which may contribute to pathogenesis of SARS 4-7. The prompt identification of ACE2 as the receptor for SARS-CoV-2 is largely due to its identification as the receptor for SARS-CoV, which emerged approximately 17 years ago. At that time, ACE2 was identified as the functional receptor for SARS-CoV after the fusion protein gene of SARS-CoV was characterized 8. Through *in vitro* studies, Li et al 9 found that: 1) ACE2 efficiently binds the S1 domain of the SARS-CoV S protein; 2) a soluble form of ACE2, but not ACE1, blocked association of the S1 domain with ACE2; 3) SARS-CoV replicated efficiently in ACE2-transfected but not mock-transfected 293T cells; and 4) anti-ACE2 but not anti-ACE1 antibody blocked viral replication on Vero E6 cells from African green monkey kidney, a cell sensitive to SARS-CoV 10, Middle East respiratory syndrome (MERS)-CoV 11, and SARS-Cov-2 infection 12. In addition, exogenous ACE2 expression allows refractory cell lines to support SARS-CoV replication 13. These results convincingly demonstrate that ACE2 is a functional receptor for SARS-CoV 9.

*In vivo* studies also consistently demonstrate that ACE2 is a crucial SARS-CoV receptor 14. A deficiency of ACE2 in mice results in a dramatic decrease in viral replication and much less severe pathologic alterations in lungs as compared to wild-type mice 14, 15. Transgenic overexpression of human ACE2 (hACE2) in mice renders them more likely to develop severe SARS phenotypes, similar to those seen in human patients 16-18. The injection of SARS-CoV spike (S) protein into mice worsens acute lung failure *in vivo* 14. Recombinant ACE2 can protect mice from severe acute lung injury by blocking SARS-CoV from binding to the membrane-bound form of ACE2 in pneumocytes, which suggests that soluble ACE2 may have a therapeutic potential for treatment of SARS 15, as well as COVID-19. Together, these extensive *in vitro* and *in vivo* results demonstrate that ACE2 serves as a major receptor for SARS-CoV infection.

### Structural analysis of ACE2 interaction with spike (S) protein of SARS-CoV-2

**Figure 2:**
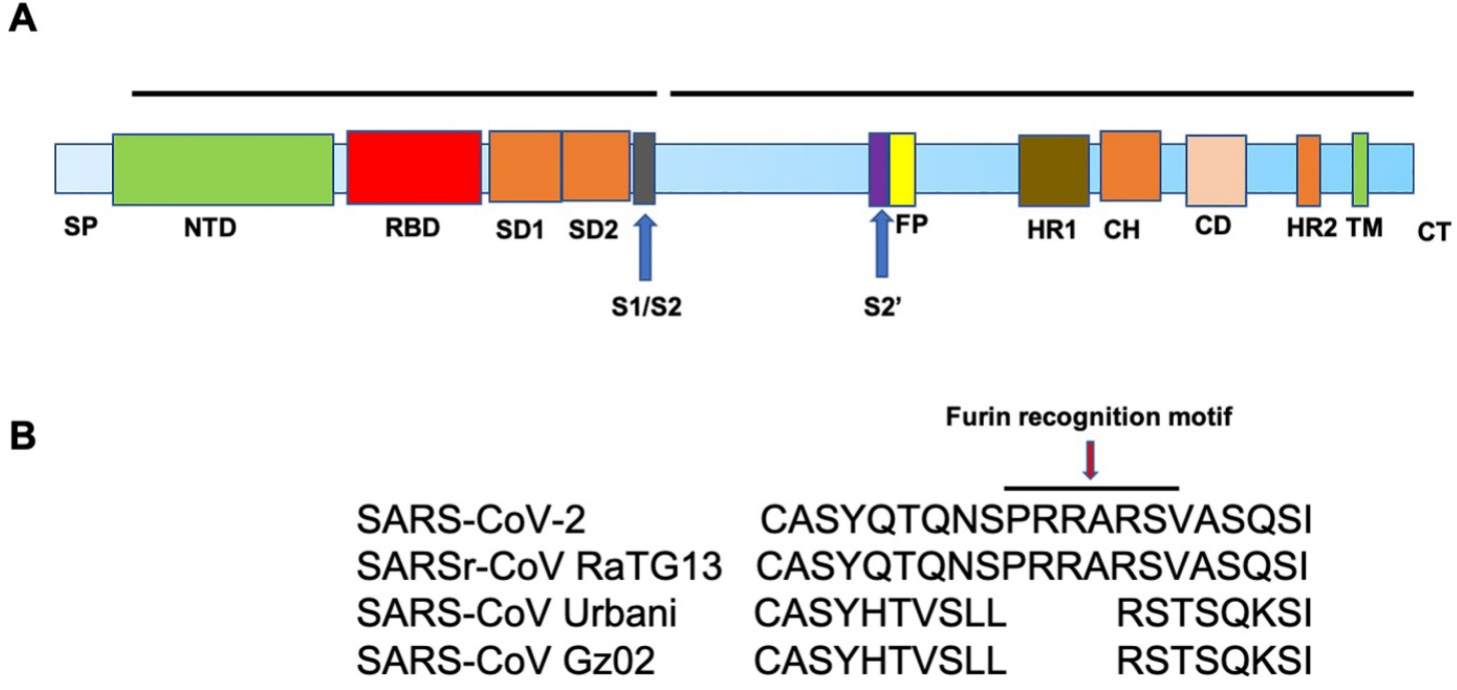
** A. Schematic representation of the structure of SARS-CoV-2 Spike protein**. The different regions on the spike are follows. SP-Signal peptide; NTD-N-terminal domain; RBD-receptor binding domain; SD1-subdomain 1; SD2-subdomain 2; S1/S2- S1/S2 protease cleavage site; S2'-S2' protease cleavage site; FP- fusion peptide; HR1-heptad repeat 1; CH- central helix; CD- connector domain; HR2-heptad repeat 2; TM-transmembrane domain and CT-cytoplasmic tail. B. Illustration of the location of the Furin cleavage site (PRRARS) in SARS-CoV-2 and SARSr-CoV RaTG13 (bat) and the absence of such sequence in SARS-CoV strains, Urbani and GZ02.

SARS-CoV-2 and SARS-CoV S proteins share 76.5% identity in amino acid sequences 19. The S protein of SARS-CoV-2 is a 1273 amino acid (aa) protein, consisting of two very important regions referred to as S1 and S2, in addition to the N-terminal 19 aa signal peptide and, in the carboxy terminal, a short transmembrane domain and a short cytoplasmic domain. The S1 region harbors the N-terminal domain (NTD) and a C-terminal domain (CTD), both of which function as the receptor-binding domain (RBD) (Figure. 2A). ACE2 serves as an entry receptor for both SARS-CoV-2 and SARS-CoV in humans via binding to their S proteins 5, 6, 20, which have almost identical 3-D structures. Recent studies from three independent groups, using Biolayer interferometry binding analysis, reported K_D_ values between ACE2 and SARS-CoV RBD of 31, 15.2, and 5.0 nM, and 15.2, 4.7, and 1.2 nM between ACE2 and SARS-CoV-2 RBD 21-23. 2.5-Å crystal structure of SARS-CoV-2 CTD complexed with hACE2 revealed the SARS-CoV-2 RBD is similar to that of SARS-CoV RBD 24. Through a separate study, using surface plasmon resonance, ACE2 was reported to bind the SARS-CoV2 S ectodomain with ~15 nM affinity, which is ~10- to 20-fold higher than ACE2 binding to SARS-CoV S 25.

### Host cell proteases are required for SARS-CoV-2 entry

Similar to SARS-CoV, SARS-CoV-2 utilizes cellular proteases, such as transmembrane serine protease 2 (TMPRSS2) 26-29 and the endosomal cysteine proteases, cathepsin B and L 30, for S protein priming to enhance virus entry 6, 28. Proteolysis of the S protein into S1 and S2 is essential for S protein-mediated CoV infection 31. Interestingly, sequence analysis of SARS-CoV-2 demonstrates the presence of four novel amino acid (PRRA) insertions between S1 and S2 21, in comparison to SARS-CoV (Figure. 2B), which results in the introduction of a cleavage site by furin, a member of the kexin-like subfamily of proprotein convertases 32. This sequence is conserved among all SARS-CoV-2 isolates sequenced to date, but is not found in the sequence of the S protein of its closest relative, RaTG13 33. In addition, the S1/S2 cleavage site of SARS-CoV-2 S harbors several arginine residues that are not present in RaTG13. The functional significance of this multibasic cleavage site remains to be ascertained. Hoffmann and co-workers demonstrated that the endosomal pH modulator, ammonium chloride, which blocks cathepsin B and L activity, strongly inhibited SARS-CoV-2 entry in TMPRSS2^-^ 293T cells 6. Furthermore, the TMPRSS2 inhibitor, camostat mesylate, partially blocked SARS-CoV-2-S entry into cells, while E-64d, an inhibitor of cathepsin B and L, in combination with camostat mesylate, fully inhibited SARS-CoV-2 S-mediated entry into cells 6.

TMPRSS2 is required for activation of a viral fusion protein but not for the S protein synthesized in and transported to the surface of cells 28. TMPRSS2 proteolytically cleaves and activates the S protein in subunit S1, which facilitates viral attachment to the surface of target cells 27, 34. TMPRSS2 is expressed in lung tissue and subsegmental bronchial branches; in the subsegmental bronchial branches, ACE2 is predominantly expressed in a transient secretory cell type 35. Colocalization of TMPRSS2 with ACE2 enhances cell entry, which correlates with TMPRSS2-mediated proteolysis of adsorbed S and ACE2 complexes 29. The significance of TMPRSS2 in mediating efficient virus infection is demonstrated in mice deficient of TMPRSS2, which have reduced body weight loss and virus in lungs following challenge with a mouse-adapted SARS-CoV 36. ADAM17, a metalloprotease can also cleave ACE2 37. TMPRSS2 was found to compete with the metalloprotease ADAM17 for ACE2 processing 37, suggesting that ADAM17-cleaved ACE2 may confer organ protection 38. Conversely, with the help of TMPRSS2, ADAM17-regulated ectodomain shedding of ACE2 may also support SARS-CoV cell entry through endocytosis, thereby contributing to infection 38. Thus, the exact role of ADAM17-mediated ACE2 shedding in SARS-CoV-2 infection of kidney and lungs requires further investigation. These findings suggest that a cell surface complex, comprising a primary receptor, ACE2, and TMPRSS2, operates as a major portal for activation of SARS-CoV 29 and SARS-CoV-2 cell entry 6.

Together, these observations demonstrate that priming of the viral S protein by host cell proteases is essential for SARS-CoV-2 entry into cells. This involves cleavage at the S1/S2 and S2′ sites by cellular protease, allowing fusion of the viral and cellular membranes and subsequent viral entry. These processes also contribute to the pathogenesis of SARS and COVID-19 39.

## Clinical and pathological features of COVID-19

Consistent with widespread expression of ACE2, several organ systems appear to be impacted by infection. Indeed, reports of new disease symptoms are disclosed almost daily; however, these may also reflect existing comorbidities or vulnerabilities of the infected individual. Typically, patients initially present with fever, chills, dry cough, and fatigue. Although less common, gastrointestinal complications, including abdominal pain, nausea, vomiting, and/or diarrhea have also been reported 40, 41. Viral RNA levels from upper respiratory specimens appear to be higher soon after symptom onset, as compared with later times 42-44. Thus, SARS-CoV-2 can be transmitted prior to the development of symptoms and throughout disease, particularly early in the course 42-44. A large number of patients first show the infection in the lower respiratory tract (possibly requiring hospitalization) with mild or no symptoms 45. SARS-CoV-2 has a mean incubation period of 5.2 days and a median duration of the onset of symptoms to death of 14 days. Patients ≥70 years of age have a shorter median duration (from the onset of initial symptoms to death) of 11.5 days, highlighting the vulnerability of this particular patient cohort 45. Among the more severe cases requiring hospitalization, leukopenia (reduced white blood cells), lymphocytopenia (reduced lymphocytes), and shortness of breath that may advance to acute respiratory distress syndrome (ARDS) and requiring mechanical ventilation are frequently observed 41, 46, 47. Bilateral lung ground-glass opacity is seen on computed tomography (CT) imaging in the majority of patients with COVID-19, consistent with pulmonary involvement 41, 48, 49, 50.

Studies focused on the clinical characteristics of infection report that patients with underlying comorbidities are more likely to progress to severe respiratory disease and even death 40, 41, 48. Among these, aging, hypertension, diabetes, and obesity appear to confer the greatest risk for the development of severe disease 41, 47, 51, 52. The majority of individuals eventually recover from infection, even among those requiring prolonged hospitalization. Concerns regarding whether patients attain full recovery prior to discharge have surfaced following anecdotal reports of a number of “cured” patients testing positive for the virus at a later date. Although it has been posited that this may reflect an absence of protective immunity among these individuals, a recent report demonstrated SARS-CoV-2 in postmortem lung acquired from a patient who had been slated for discharge prior to succumbing to sudden cardiac arrest 53. Interestingly, this individual had three consecutive SARS-CoV-2 PCR negative nasopharyngeal swabs and improved significantly, indicating she had recovered from the infection. Immunohistochemistry and electron microscopy, however, revealed residual virus in the lung, and histopathological investigation showed diffuse alveolar damage consistent with lung injury previously observed in patients with SARS and MERS 53. The findings from this postmortem investigation yield important insight into SARS-CoV-2/COVID-19 pathogenesis and argue against the idea that previously infected individuals remain at risk for a second SARS-CoV-2 infection. This remains a significant clinical and public health concern that requires further investigation.

Although lung appears to be the primary site of infection and injury, other tissues are also impacted by COVID-19, particularly the vascular system. A comprehensive autopsy study 54 revealed deep vein thrombosis in 7 of 12 patients (58%) studied, in whom venous thromboembolism was not suspected before death. In the same investigation, pulmonary embolism had been the direct cause of death in 4 patients. Consistently, postmortem examinations reveal diffuse alveolar damage with severe capillary congestion and variegated findings in lung and other organs, suggesting COVID-19-related endothelial cell infection, endotheliitis, and vascular dysfunction 55-58. Postmortem investigation of tissues that may harbor virus demonstrated that high levels of SARS-CoV-2 RNA in lung of all 12 patients examined, while 5 of the 12 patients demonstrated high viral titers in liver, kidney, or heart 54. These autopsy observations indicate that the pathogenesis of severe COVID-19 involves significant coagulation and platelet activation, endothelial cell and severe ongoing lung damage, and other organ failure. Although clinical observations and autopsy studies greatly help us to understand the pathogenesis of COVID-19, the mechanism underlying COVID-19 pathogenesis still require further experimental studies.

## Pathogenic roles of ACE2 in the infection and pathogenesis of COVID-19

Previous recognition of the role of ACE2 in SARS pathogenesis greatly facilitates our understanding of the pathogenesis of COVID-19. In this section, we will review the function of ACE2 and the RAS and the potential pathogenic role of ACE2 in SARS.

### Function of ACE2 and the renin-angiotensin system (RAS)

RAS consists of renin, angiotensin, and aldosterone. RAS plays an important role in regulating blood volume and systemic vascular resistance, which together influence cardiac output and arterial pressure 59-61. ACE2 is a peptidase of the RAS, a primary cardiovascular regulatory system 59-61. ACE, a dipeptidyl carboxypeptidase in the RAS, converts the inactive decapeptide Ang I into the active octapeptide and potent vasoconstrictor Ang II (Figure 1) and inactivates the vasodilator bradykinin 62. ACE2 largely counteracts the activity of ACE by degrading Ang I into the nonapeptide, Ang 1-9, an inactive form of angiotensin. It can also degrade and hydrolyze the vasoconstrictor, Ang II, into a heptapeptide, Ang (1-7), which acts as a potent vasodilator 59-61. Ang II, a peptide with multiple actions, promotes cardiovascular diseases (CVD), which can be antagonized by the effects of Ang (1-7) 63, 64. It is important to note that the conversion of Ang II to Ang (1-7) is the primary function of ACE2 and kinetic studies reveal that Ang II is a much better substrate for ACE2 than Ang I 65 (Figure 1). The effects of Ang II are largely mediated through angiotensin II receptor type I (AT1), which is opposed by Ang (1-7) 63 (Figure 1). ACE breaks down Ang (1-7) into Ang 1-5, the major degrading pathway for Ang (1-7) 63.

Ang II is responsible for most of the physiological and pathophysiological effects of the RAS (Figure 1). Inhibitors of ACE that reduce the formation of Ang II have been successfully applied as an effective hypertension treatment (Figure 1). ACE inhibitors are also a standard therapy following myocardial infarction to delay the development of heart failure and reduce the progression of renal disease (Figure 1). ACE2 influences ACE by altering the ratio of Ang II and Ang (1-7), effectively modulating the balance between vasoconstrictors and vasodilators within the heart and kidney 66-68 (Figure 1). Human ACE2 has some homology (40% identity and 61% similarity at the amino acid level) to human ACE 69. Despite sharing significant homology and many biochemical properties with ACE, ACE2 activity cannot be blocked by classical ACE inhibitors 70.

### ACE2 expression pattern and factors influencing ACE2 expression level

The ACE2 gene contains 18 exons that map to chromosome Xp22. Thus, gender differences in the RAS, CVD, and renal diseases may be linked to the ACE2 gene 60, 69. Both human and mouse ACE2 contain 805 amino acids that include an N-terminal signal sequence, a single active-site catalytic region, and a C-terminal hydrophobic membrane-anchor region 70. ACE2 is expressed predominantly by epithelial cells of the lungs and kidneys, and cardio myocytes 66, primary targets of SARS-CoV and SARS-CoV-2 64. scRNA-seq data analyses recently revealed that ACE2 is highly expressed in organs at risk for injury in the context of COVID-19 and SARS, including lung, heart, esophagus, kidney, bladder, ileum, oral mucosa, and specific cell types (e.g., type II alveolar cells (AT2) in lung, myocardial cells, proximal tubule cells of the kidney, ileum and esophagus epithelial cells, and bladder urothelial cells) 42, 71. ACE2 protein expression is high in renal and cardiovascular tissues and much of the gastrointestinal tract, particularly ileum, duodenum, jejunum, caecum, and colon 2. Interestingly, ACE2 expression is regulated by aging and genetic alteration, which may explain the varying susceptibility among different ages and individuals. One study showed no gender-related differences in ACE2 expression between young and middle-aged adults, however, greater ACE2 expression is observed between young adults those of advanced age, which may contribute to the predominance in SARS-CoV-2 infection and development of more severe disease among older individuals 72. A more recent study, however, does not support this finding 73. Car, et al analyzed four large-scale datasets of normal lung tissue and found that race, age, or gender do not appear to influence ACE2 gene expression 73. This study did reveal significantly higher ACE2 gene expression in lung of smokers compared to non-smokers, suggesting that smokers may be more susceptible to COVID-19 73. Genetic alterations in the expression of ACE2 develop a diverse pattern of phenotypes, ranging from hypertension and metabolic and behavioral dysfunctions to impairments in serotonin synthesis and neurogenesis 74, 75.

The level of ACE2 is also influenced by some CVD and antihypertensive drugs targeted to RAS, such as Ang II receptor blockers (ARBs), but not ACE inhibitors (Figure 1) 76. Plasma ACE2 activity is low in healthy subjects but elevated in patients with CVD 77. ARBS, such as olmesartan, but not ACE inhibitors, increase urinary ACE2 76. This potentially offers additional renoprotective effects in patients who use olmesartan 76. ACE inhibitors increase Ang (1-7) through Ang I conversion by neprilysin so altered ACE2 is not required 63. Increased Ang II levels are suggested to upregulate ACE2 activity 60. In ACE2-deficient mice, Ang II levels are approximately double that of wild-type mice, while Ang 1-7 levels are almost undetectable 60. Loss of ACE2 accelerates diabetic kidney injury in these mice and contributes to the late development of Ang II-dependent glomerulosclerosis 78, 79. Using adeno-associated virus (AAV) or adenoviral-mediated gene delivery of ACE2 or Ang (1-7) protects against diabetes-induced retinopathy and glomerular injury in rodent models 80-82. Further, targeting the degradation of Ang II with recombinant ACE2 prevents angiotensin II-dependent hypertension 83 and human recombinant ACE2 reduces the progression of diabetic nephropathy 84. Together, these findings suggest that ACE2 plays a crucial role in the RAS because it opposes the actions of Ang II 60, 69 (Figure 1). Moreover, ACE2 has a beneficial role and therapeutic potential in many diseases such as hypertension, diabetes, and cardiovascular disease. Because ACE2 performs these vital functions, as well as serving as a receptor for SARS-CoV-2 infection that result in its downregulation, it is conceivable that ACE2 plays a key role in the pathogenesis of SARS and COVID-19.

### ACE2 in pathogenesis of SARS

SARS-CoV S protein downregulates ACE2 expression and thereby promotes lung injury 85. This downregulation effect may result from enhanced shedding/internalizing processes and other unknown mechanisms 85, 86. Consistently, SARS-CoV replicated efficiently in ACE2-positive Vero cells, which also reduced ACE2 expression, indicating robust receptor interference in the context of SARS-CoV 14. Since a virus must dock and enter the cells before it can replicate, surface localization of ACE2 and the state of cell differentiation may have a strong impact on SARS-CoV disease and other pathologies triggered by acute lung injury 75. As discussed above, the maintenance of normal ACE2 levels in lung is beneficial for combatting inflammatory lung disease 66-68. The RAS acts both systemically and locally in a variety of tissues, including the lung 75. These results suggest a molecular mechanism for SARS-CoV-associated severe and often lethal lung failure and support a potential therapeutic strategy for preventing or attenuating SARS and COVID-19 14, 75.

### Potential pathogenic role of ACE2 in COVID-19

Although ACE2 is widely recognized as the primary receptor for SARS-CoV-2, its role in the pathogenesis of COVID-19 remains elusive. ACE2 plays an essential role in virus infection and dissemination; however, whether ACE2 contributes to the progression of severe COVID-19 through additional means remains unclear. SARS-CoV-2 infects ACE2-expressing cells in the lung, leading to shedding of ACE2 from tissue, effectively reducing the level of ACE2 expression by infected cells. On the other hand, if ACE2 is shed into the bronchial space by SARS-Cov-2, the peptidase is still active and can cleave Ang II to Ang (1-7). Within the bronchial space, ACE2 can still bind SARS-CoV-2 but doesn't internalize, since it is no longer attached to the apical membrane of the epithelial cells (Figure 1). Whether this shedding prevents other cells from SARS-CoV-2 infection remains elusive and warrants further investigation.

In a mouse model, membrane-bound ACE2 was demonstrated to play a critical role in anti-inflammation through RAS signaling and conversion of Ang II to Ang (1-7), which protected animals against acute lung injury 15. It is conceivable that the reduction of ACE2 expression may promote inflammation in the lung and the subsequent cytokine storm that presents in many severe COVID-19 patients (Figure 1). ACE2 maintains proper function of heart and kidney and downregulation of ACE2 by SARS-CoV-2 may compromise this protective feature and contribute to the damage caused by infection of these organs (Figure 1). Since ACE2 also serves as a binding site for SARS-CoV-2, enriched expression in lung and other tissues may play a dual role in facilitating virus entry (detrimental) and protection from additional viral attack (protective) (Figure 1), depending on the stage of infection 87, 88. Of note, ACE2 has collectrin domains that facilitate amino acid transport. There is 47.8% identity between ACE2 collectrin domains and Tmem27 (collectrin) 89. Since Tmem27 is expressed in multiple tissues including guts and lacks the extracellular peptide domain of ACE2, it is not expected to directly bind and enable internalization of SARS-CoV-2^64^, ^90^. Animal studies indicate that angiotensin-converting enzyme inhibitors (ACEIs) and angiotensin receptor blockers (ARBs) may increase ACE2 expression. Patients who take ACEIs and ARBs were assumed to be at increased risk for SARS-CoV-2 infections due to elevated ACE2 expression 91, 92. Use of ACEIs were thought to cause increased morbidity and mortality of COVID-19 93, however, this hypothesis is not supported by other clinical findings, where recent studies show that ACEIs/ARBs are unrelated to the severity or mortality of COVID-19 in such patients 94, 95 and may even improve the outcome of COVID-19 as discussed extensively below 96. These clinical studies suggest that the downregulation of ACE2 caused by SARS-CoV-2 infections may accelerate the progression of COVID-19 from mild to severe disease via increased activity of the RAS (Figure 1).

Many patients with COVID-19 present with dysfunction of multiple organs including heart, kidney, and intestine, which are also rich in ACE2 expression. It is still unclear whether the damage is from direct infection of ACE2-expressed endothelial/epithelial cells at those tissues, or an indirect effect from the whole-body cytokine storm in severe COVID-19 patients. Enriched ACE2 expression in endothelial/epithelial cells might make these tissues vulnerable to the virus entering the circulation system from the lung. SARS-CoV-2 infection of kidney tubules and the digestive system has been documented through biopsy studies 97, 98. This may result in renal injury/failure and digestive disorders, such as loss of appetite and diarrhea experienced by some infected individuals 97, 98. Cardiovascular complications are rapidly emerging as a key threat in COVID-19, in addition to respiratory, renal and digestive diseases 99. Recent findings show the presence of viral elements within endothelial cells and an accumulation of inflammatory cells, in addition to endothelial and inflammatory cell death 99. These findings suggest that SARS-CoV-2 infection facilitates the induction of endotheliitis in several organs as a direct consequence of viral involvement (as noted by the presence of viral bodies) and of the host inflammatory response 99. COVID‐19 patients also exhibit some neurological manifestations, including loss of smell, ataxia, and convulsions, indicative of a potential direct or indirect attack on neurons and/or glia 51, 100. It is likely that disease exaggeration up to the late stage may be attributed not only to direct viral damage, but also generalized hypoxia and/or immune-mediated injury induced by SARS-CoV-2 101. Two pathological features occur in severe COVID-19 patients: progressive increase of immune activation and cytokine storm, and an unusual trend of hypercoagulation and platelet activation 101. However, whether ACE2 expression on cells of multiple organs contribute to multi-organ damage in patients is unclear, as does the role of ACE2 in immune activation and cytokine storm. Whether downregulation of ACE2 in the infected organs facilitates multiple organ failure also remains unclear.

A recent study using an engineered human tissue system demonstrated that soluble human ACE2 can reduce viral growth *in vitro* and inhibit SARS-CoV-2 infection in blood vessels and human kidney organoids on the early stages of infection 102, suggesting exogenous ACE2 could block early entry of SARS-CoV-2. Further, because of the protective effects of ACE2 on chronic underlying diseases and ARDS, the development of therapeutic enhancers of ACE2 activity, such as diminazene aceturate (DIZE) 103, which are currently used as antiparasitic drugs, has been proposed for the treatment of COVID-19 patients in the late stages of disease 104. However, due to lung, gut, liver and kidney toxicity, it is very unlikely to enter clinical trial 105, 106. Further, whether or not administration of soluble ACE2 or ACE2 activator is beneficial in later stages of the disease requires further study. ACE2 gene polymorphisms do not affect the outcome of severe ARDS 107 and results obtained from human studies of the role of ACE2 gene polymorphisms in human hypertension are inconclusive. Some studies suggest that the gene polymorphism is association with human hypertension 108-115, which is not supported in other studies 116. The role of ACE2 in the development of COVID-19 is complex. It may serve as a binding target, but it may also directly participate in the pathogenesis of disease and contribute to immune activation and dysfunction. Further investigation in animal models and clinical trials is much needed to clarify these mechanisms.

## Animal models for pathological and preclinical studies of COVID-19

An ideal animal model for pathogenesis and preclinical studies should reflect the clinical signs, viral replication, and pathology seen in humans 117. Pathologically, the model should show severe COVID-19 symptoms such as severe pneumonia, multiple organ failure, and even death, all of which are essential for pathogenesis studies. The animal models for COVID-19 will also facilitate the development of a vaccine and therapeutics for prevention and treatment of COVID-19, although they do not need to replicate all aspects of disease to provide useful information for preclinical studies 118. For these reasons, many labs are rushing to develop COVID-19 models in various species and transgenic animals 119. Considering SARS-CoV and SARS-CoV-2 utilize the same hACE2 receptor for entry into the host, leading to SARS and COVID-19 in humans, we will review animal models for both SARS and COVID-19.

### Animal models for SARS

No single model offers a direct reproduction of what is seen in humans with SARS 118, 120, 121. In the spontaneous condition without molecular engineering, monkeys, cats, ferrets, mice, swine, chickens, hamsters, guinea pigs, and rats have been experimentally infected 117, 122-124. Most display mild signs with severe acute pneumonia-like symptoms after infection, and show SARS-CoV replication in their serum followed by spontaneously clearing the virus 117, 122, 123. Some studies show that ferrets exhibit the strongest clinical symptoms and pathology 120, 123, 125; however, these findings were not reproduced in later studies 121, 123, 126. Studies conducted in various species of NHPs such as rhesus macaques, cynomolgus macaques, and African green monkeys support SARS-CoV replication, and the animals develop pneumonitis with variable mild clinical symptoms, depending upon the species employed 120, 127. No single NHP species is highly preferred at this time 120.

Although mice are the best model for pathogenesis studies of SARS, inbred mouse strains such as B6, BALB/C, and 129 mice infected with SARS-CoV do not show associated signs of clinical illness or overt pathology that are reproducible and equivalent in severity to those observed in SARS patients 118. In addition, SARS-CoV infects old BALB/C wild type mice (12-14 months old) and shows some severe phenotypes 128, while SARS-CoV-infected aged BALB/c mice demonstrate signs of clinical illness characterized by significant weight loss, hunching, ruffled fur, and slight dehydration measured by skin turgor 128. Although this age-related increase in morbidity in BALB/c mice represents the clinical characteristics seen in humans in the 2003 SARS outbreak, as well as the current SARS-CoV-2 crisis, in which age is a significant risk factor for severe disease and poor outcome, old mice are very difficult to procure for experimental studies and their immune senescence also complicates pathogenesis studies 118, 129.

### Development of mouse-sensitive SARS-CoV strains

To compensate for the disadvantage of using old mice, a mouse-sensitive SARS-CoV derived from the Urbani strain by serial passage in the respiratory tract of young BALB/c mice has been developed 129. Fifteen passages resulted in a virus (MA15) that is lethal for mice following intranasal inoculation 129. Lethality is preceded by rapid and high titer viral replication in lungs, viremia, and dissemination of virus to extrapulmonary sites accompanied by lymphopenia, neutrophilia, and pathological changes in the lungs 129. Abundant viral antigen is extensively distributed in bronchial epithelial cells and alveolar pneumocytes, and necrotic cellular debris is present in airways and alveoli, with only mild and focal pneumonitis 129. These observations suggest that mice infected with MA15 die from an overwhelming viral infection with extensive, virally mediated destruction of pneumocytes and ciliated epithelial cells 129. The MA15 virus has six coding mutations associated with adaptation and increased virulence; when introduced into a recombinant SARS-CoV, these mutations result in a highly virulent and lethal virus (rMA15), duplicating the phenotype of the biologically derived MA15 virus 129. Intranasal inoculation with MA15 reproduces many aspects of disease seen in severe human cases of SARS. Another new strain of SARS-CoV was further derived from serially passaged 25 times in BALB/c mice at 3-day intervals and resulted in a highly lethal strain designated as v2163 130. This v2163 strain was more severe, with higher lethality even in 5- to 6-week-old BALB/c mice than MA15 strains. It had nine mutations affecting 10 amino acid residues. Strain v2163 increased interleukin (IL)-1α, IL-6, macrophage inflammatory protein (MIP)-1α, monocyte chemoattractant protein (MCP)-1, and regulated on activation, normal T cell expressed and secreted (RANTES) in mice, with high IL-6 expression correlating with increased mortality 130. The infection largely mimicked human disease, but lung pathology lacked hyaline membrane formation 130. Nonetheless, these strains may be very helpful for pathogenesis studies 130. However, since the strains are mutated to be sensitive to mice but not humans, there are some concerns regarding the clinical relevance obtained by using these strains for preclinical studies in mice.

### hACE2 transgenic lines

To compensate for this drawback, transgenic overexpression of hACE2 in mice may serve as a viable alternative for SARS and COVID-19 models 16-18. Introduction of hACE2 to mice renders them highly sensitive to the development of SARS, as seen in human subjects 16-18. Yang and co-workers developed a mouse model of human SARS-CoV infection by introducing hACE2, driven by the mouse ACE2 promoter, into the mouse genome 16. Mice infected with a human SARS strain show pulmonary lesions, including interstitial hyperemia and hemorrhage, monocytic and lymphocytic infiltration, protein exudation, alveolar epithelial cell proliferation and desquamation, and even death 16. Additionally, SARS-CoV replicated more efficiently in the lungs of transgenic mice than in those of wild-type mice. Other pathologic changes, including vasculitis, degeneration, and necrosis, were found in the extrapulmonary organs of transgenic mice, and viral antigen was found in brain 16. Two other transgenic mouse models that express hACE2 specifically in the airway and other epithelia, or systemically in multiple tissues and cells, develop a rapidly lethal infection after intranasal inoculation with SARS-CoV 17, 18. Infection begins in airway epithelia, with subsequent alveolar involvement and extrapulmonary virus spread to the brain 17. Infection results in macrophage and lymphocyte infiltration in the lungs and upregulation of proinflammatory cytokines and chemokines in both the lung and the brain 17. Further, another rapid model for SARS and COVID-19 can be achieved by over-expressing hACE2 in lungs of wild type B6 mice by intranasally administering adenovirus 5-expressing hACE2 (*Ad5-hACE2*). An advantage of this model is that it can be easily adapted for performing pathogenesis studies in any given molecular-engineered mouse lines without multiple mouse crosses. This approach has been successfully used to generate a rapid mouse model for MERS via instilling an Ad5 vector encoding MERS receptor, hDPP4, and prior to MERS-CoV infection 131.

### Animal models for COVID-19 for pathogenesis and preclinical studies

Some studies indicate that mice, cats, hamsters, ferrets, and nonhuman primates can be infected by SARS-CoV-2 132. After infection, however, only mild phenotypes in lung are seen that do not progress to severe disease. A recent study demonstrates that ferrets and cats are highly susceptible to SARS-CoV-2, while dogs have low susceptibility and livestock, including pigs, chickens, and ducks are not susceptible to the virus 132. However, SARS-CoV-2 only replicates in the nasal turbinate, soft palate, and tonsils of ferrets, which is not comparable with SARS-CoV, which replicates in both the upper and lower respiratory tract of ferrets 132. Another study reported that Syrian hamsters infected by SARS-CoV-2 developed mild to moderate clinical syndrome and mild lung pathology changes 133. The maximal clinical signs included rapid breathing, weight loss, histopathological changes from the initial exudative phase of diffuse alveolar damage with extensive apoptosis to the later proliferative phase of tissue repair, airway and intestinal involvement with virus nucleocapsid protein expression, high lung viral load, and spleen and lymphoid atrophy associated with marked cytokine activation, all of which were observed within the first week of virus challenge 133. Challenged index hamsters consistently infected naïve contact hamsters housed within the same cage, resulting in similar pathology but not weight loss 133. All infected hamsters recovered and developed mean serum neutralizing antibody titer ≥1:427 fourteen days post-challenge. Immunoprophylaxis with early convalescent serum achieved a significant decrease in lung viral load but not in lung pathology 133. Several laboratories across the world have shown with high reproducibility that rhesus macaques are infectable with SARS-CoV-2, showing evidence of virus replication and shedding in nasal swabs with mild clinical signs 119. A very recent study by SARS-CoV-2-infected rhesus macaques show SARS-CoV-2 infection protects against rechallenge 134, indicating that immunologic approaches to the prevention and treatment of SARS-CoV-2 infection may be possible 134. Other nonhuman primate models for COVID-19 are currently being developed by several labs, including those at the Tulane National Primate Research Center.

Using hACE2 transgenic mice, Bao et al documented that mice overexpressing hACE2 were infected with SARS-CoV-2. The infected mice showed weight loss and virus replication in lung with mild to moderate changes in lung pathology 135. The typical histopathology was interstitial pneumonia with infiltration of significant lymphocytes and monocytes in alveolar interstitium, and accumulation of macrophages in alveolar cavities. Viral antigens were observed in bronchial epithelial cells, alveolar macrophages, and alveolar epithelia. This phenomenon was not found in wild type mice with SARS-CoV-2 infection 135. The pathogenicity of SARS-CoV-2 in hACE2 mice was clarified and the Koch's postulates were fulfilled 135. Although it does not develop severe clinical manifestations seen in severe COVID-19, this mouse model will facilitate our understanding the pathogenesis of COVID-19. Together with the SARS-CoV-2-infected nonhuman primate, ferret, cat, and hamster models, mouse models will be very useful for the development of therapeutics and vaccines against SARS-CoV-2. However, they require further optimization, including conducting experiments in old mice or mice with induced pre-existing conditions such as hypertension, obesity, diabetes, and respiratory deficits, as seen in human patients, or with immune compromised or conditionally induced-immune cell depleted conditions 136-140.

In summary, there are no perfect animal models that develop severe COVID-19 phenotypes as seen in severe human COVID-19. There is a pressing need to develop animal models that show reproducible and severe COVID-19 symptoms for pathogenesis studies of the role of ACE2 in COVID-19 and preclinical studies of potential therapeutics and vaccines.

## Therapeutic approaches

Currently there is no effective antiviral therapy, therefore prevention or treatment of COVID-19 can be an alternative approach to mitigate rapid disease progression and high mortality. Clinical trial studies targeting ACE2/Ang 1-7 axis and ACE2 interaction with the S protein is drawing a great attention for treating COVID-19 patients with lung and cardiovascular damage and preventing the spreading of the disease 141. Also, these clinical trial studies will greatly facilitate the pathogenesis of COVID-19. Therefore, we will review the current therapeutic and vaccine developments related to blocking ACE2 and S protein interactions and inhibiting RAS, anti-SARS-CoV-2 drugs, and immune-based therapy.

### Spike and ACE2 interaction inhibitors

The spike (S) protein mediates viral receptor-binding and membrane fusion. As such, inhibitors that prevent either receptor binding or membrane fusion may serve as viable strategies for preventing further virus dissemination in infected patients. Specifically, disruption of the interaction between ACE2 and the critical motifs within the S2 subunit, consisting of a fusion peptide (FP) region and two heptad repeat regions: HR1 and HR2 (Figure 2) may be particularly effective. In this regard, a pan-coronavirus fusion inhibitor peptide, EK1, targeting the HR1 domains of HCoV S proteins, has been proven to be effective against SARS-CoV-2 S protein-mediated membrane fusion in vitro 142. The same group has also shown that a lipopeptide version of EK1 known as EK1C4 prevented SARS-CoV-2 S protein-mediated membrane fusion and pseudovirus infection with IC50s of 1.3 and 15.8 nM. This inhibitory effect is 241- and 149-fold more potent than the original EK1 peptide, respectively 143. With regards to spike protein and ACE2 receptor interaction, an alternative approach is to mop up the virus using high concentrations of APN01, a recombinant soluble form of human ACE2 (rhACE2) to not only block viral entry but also protect lungs from injury. It has been reported that S protein from SARS-CoV does not influence the hydrolysis of Ang II to Ang-(1-7) by soluble ACE2 144. Therefore, spike inhibitors may not bind and downregulate ACE2. Ang II or other peptide substrates would directly interfere with SAR-CoV-2 binding and internalization. A clinical trial assessing effects of rhACE2 in COVID19 patients was proposed in China but was later withdrawn (NCT04287686) 64. However, APN01 (rhACE2), developed by APEIRON, is slated for a multicenter trial in Europe (NCT04335136). It is important to note, that giving ACE2 in the circulation would not necessarily prevent SARS-2 infection. Instead, an intranasal delivery of rhACE2, would likely increase the efficacy for prevention and treatment of COVID-19, which warrants further clinical trial studies. Some other potent recombinant ACE2 drugs such as ACE2-Ig that has a better pharmacological property and a potent neutralizing effect in SARS-CoV-2 pseudotyped virus is under the development for the countermeasure of COVID-19 145.

### Neutralizing antibodies

In contrast to vaccines, since an antibody offers immediate protection, development of an antibody that binds to a conserved epitope on the spike protein of SARS-CoV-2 can neutralize the virus in patients even before they produce their own antibodies. Following studies thus far have been successful in generating neutralizing antibodies targeting the S protein that are effective *in vitro*. Single domain antibodies (VHHs) from a llama immunized with prefusion-stabilized coronavirus S protein neutralizes SARS-CoV-2 S pseudotyped viruses 146. A human monoclonal antibody mAb 47D11 that most likely targets the conserved core structure of the S1B RBD, neutralizes SARS-CoV-2 in cell culture 147. A cross-neutralization antibody S309, potently neutralizes SARS-CoV-2 and SARS-CoV pseudoviruses as well as authentic SARS-CoV-2 by engaging the S RBD 148. Spike-specifc monoclonal antibodies derived from single B cells of SARS-CoV-2 infected individuals have been isolated and characterized 149. These neutralizing antibodies will be tested for preventing the spread of COVID-19 and treating COVID-19 severe patients 148. Further, convalescent plasma therapy for COVID-19 is viable approach to reduce COVID-19 mortality since it appears safe, and clinically effective. In this regard, several studies showed that convalescent plasma obtained from recently recovered COVID-19 patients improved the clinical outcomes by neutralizing viremia in severe COVID-19 cases if started early but not in critically ill patients 150-153.

### Immune-Based Therapy

The amplified immune response and cytokine release or “cytokine storm” seen in SARS-CoV-2 infection could result in multiorgan failure. Therefore, monoclonal antibodies directed against inflammatory cytokines or its receptors can be adjunctive therapies for COVID-19. In this regard, clinical studies show increased IL-6 to be a key driver of inflammation 7. Several randomized clinical trials with Tocilizumab (monoclonal antibody against IL-6), alone or in combination (NCT04310228, ChiCTR20000297), and Sarilumab, another IL-6 receptor antagonist (NCT04315298) are underway in patients with COVID-19 worldwide.

### Vaccines

There is an unprecedented need to develop effective COVID-19 vaccine. To meet this challenge there is a world-wide multipronged strategic approach for vaccine development around the globe. In this effort, just released clinical data from phase I study of mRNA-1273, vaccine candidate encoding for a perfusion-stabilized form of the SARS-CoV-2 spike protein, report that patients dosed at 25 µg or 100 µg had seroconverted by day 15 after a single dose. mRNA-1273 vaccination resulted in high neutralizing antibody titers in eight patients that exceeded levels seen in convalescent sera (National Institute of Allergy and Infectious Diseases (NIAID) and Moderna).

In a recent study, DNA vaccine candidates expressing different forms of the SARS-CoV-2 Spike (S) protein was evaluated in rhesus macaques. Vaccinated animals developed humoral and cellular immune responses and neutralizing antibody titers comparable to those found in convalescent humans and macaques infected with SARS-CoV- 2 154.

The other strategy involves using Ad5-nCoV, a recombinant vaccine incorporating the adenovirus type 5 vector by CanSino, which has received approval from the Chinese authorities to begin human trials. Inovio Pharmaceuticals in collaboration with Beijing Advaccine Biotechnology is developing a DNA vaccine against COVID-19 called INO-4800. Development of a heterologous SARS-CoV RBD recombinant protein has been proposed to be a vaccine approach potential against COVID-19 155.

### Angiotensin converting enzyme inhibitors (ACEI) and angiotensin receptor blockers (ARBs)

Hypertension is a common risk factor for mortality among individuals with COVID-19. It is postulated that the angiotensin system modulation by ACEI, such as enalapril, lisinopril, captopril, and ramipril, or ARBs, such as losartan, candesartan, and valsartan, could be used in prevention and treatment of SARS-CoV-2. The use of these two angiotensin system modulators in clinical management of COVID-19 has taken center stage due to controversial findings in animal studies that show increase in ACE2 expression with use of the ACEI, lisinopril, or ARBs, losartan, at doses that are much higher than that prescribed to patients 156. Inpatient use of ACEI/ARBs is associated with a lower risk of all-cause mortality and improvement of clinical outcomes compared with ACEI/ARBs non-users 157, 158. Treatment with ACEIs or ARBs continues to provide cardiovascular and renal protection in patients diagnosed with COVID-19 157, 158, so discontinuing these medications may be harmful in this patient population 159. Therefore, several scientific societies highly recommend that patients continue their current hypertensive medication regimen as we await the results of randomized controlled trials addressing the impact of renin-angiotensin system inhibitors (RASIs) on COVID-19 morbidity and mortality 160. For example, the current Centers for Disease (CDC) Control and Prevention guidance for clinical care of patients with COVID-19 (April 3, 2020) highlights that “…there are no data to suggest a link between ACEIs or ARBs with worse COVID-19 outcomes”. The American Heart Association (AHA), the Heart Failure Society of America (HFSA), and the American College of Cardiology (ACC) recommends continuation of these drugs for patients already receiving them for heart failure, hypertension, or ischemic heart disease (HFSA/ACC/AHA Statement Addresses Concerns Re: Using RAAS Antagonists in COVID-19).

It is important to note that data from human studies show no increase ACE2 expression by ACEI or ARBs 161-163. A caveat of ARB or ACEi effects on ACE2 expression in human is that only the shed form of ACE2 is studied in plasma or urine where ACE2 content is quite low and local tissue concentration of ACE2 are unknown and require further investigation. Very recent study shows that ACEI offers greater protection than ARBs in COVID-19 patients 164.

Since hyper-inflammation state seen in COVID-19 cases, can be a result of angiotensin peptide (1-7) deficiency (Figure 1), a non-randomized interventional clinical trial to evaluate the possible effect of plasma derived angiotensin peptide (1-7) supplementation on treatment of COVID-19 cases (NCT04375124) is in progress. To examine the therapeutic values of ACEI and ARBs in COVID-19 patients, several clinical trial studies have recently launched. Two randomized clinical trials examining the role of losartan in COVID-19 in USA (NCT04312009, NCT04311177) is underway. A double-blind, placebo-controlled 1:1 randomized clinical trial to assess if ARB valsartan may prevent the development of ARDS and avert morbidity (admission to intensive care unit (ICU) and mechanical ventilation is initiated in Netherland (NCT04335786).

### Antiviral drugs

Existing antiviral drugs are effective in treating related, lopinavir-ritonavir, and hydroxychloroquine and chloroquine used in patients during SARS or MERS outbreak, to assess their effectiveness in inhibiting SARS-CoV-2 replication.

#### Remdesivir

Remdesivir is an inhibitor of the RNA-dependent RNA polymerase. To date, studies on remdesivir show that is highly effective in inhibiting 2019-nCoV infection in Vero E6 cells 165, 166. In a recent study compassionate-use of remdesivir in patients hospitalized for severe COVID-19 showed clinical improvement in 36 of 53 patients 167. In a randomized, double-blind, placebo-controlled, multicenter trial in severe COVID-19 patient Hubei, China showed no statistically significant clinical benefit 168. Preliminary results from a randomized, controlled clinical trial with remdesivir indicate that critically ill COVID-19 patients who were given intravenous infusion of remdesivir had a 31% faster time to recovery than those who received placebo. The median time to recovery was 11 days for patients treated with remdesivir compared with 15 days who were in placebo group (NCT04280705).

#### Lopinavir-Ritonavir

Lopinavir was shown to have antiviral effect against SARS-CoV-2 virus in Vero E6 cells 166. A study with lopinavir/ritonavir showed that it does not shorten the duration of SARS CoV-2 shedding 169, however, in a study from Hong Kong, compared with ribavirin alone, patients treated with lopinavir/ritonavir plus ribavirin had a lower risk of ARDS or death caused by SARS-CoV after the onset of symptoms 170.

#### Hydroxychloroquine and Chloroquine

Chloroquine (CQ) and hydroxychloroquine (HCQ) are aminoquinolines, which have been used to treat malaria and autoimmune diseases. Recent studies show that CQ and HCQ is effective against SARS-CoV-2 infection in Vero E6 cells 165, 171. However, a prospective randomized trial of 30 adults with COVID-19 in China revealed that HCQ with conventional treatment were compared with 15 patients treated with conventional treatment only showed no effectiveness of HCQ, and one patient in the HCQ group progressed to severe disease 172. An open-label non-randomized clinical trial showed that azithromycin added to HCQ was significantly more efficient for virus elimination 173. The outcome of a randomized, double-blind, phase IIb clinical trial (CloroCovid-19 Study) showed that at two different CQ doses as adjunctive therapy for SARS-CoV-2 patients, patients on high dose CQ arm presented more QTc > 500 ms (25%), and higher fatality (17%) than the lower dosage in the first recruited 81 patients 174. In a recent observational study assessing the effects of chloroquine or hydroxychloroquine, with or without a macrolide, in 96032 hospitalized COVID-19 patients indicate any benefit of 4-aminoquinoline-based treatments in this population and suggest that they could even be increased risk of de-novo ventricular arrythmia 175 These data prove that HCQ or CQ has no therapeutic role either in the treatment or in the prophylaxis of COVID-19.

### Advantages and Limitations of therapeutics

SARS-CoV-2 continues to spread globally without any approved therapies and researchers are urgently searching for effective prophylactic and therapeutic interventions. Therefore, repurposing existing drugs approved for other uses could greatly benefit COVID-19 patients around the world despite benefits and limitations. As an example, in vitro studies using drugs such as lopinavir/ritonavir, HCQ and CQ, and remdesivir have shown effectiveness against SARS-CoV-2 however, small non-randomized trials using these drugs alone or in combination have shown mixed results. In the context of convalescent plasma therapy and vaccines, earlier studies have shown that high concentrations of anti-sera against SARS-CoV neutralized SARS-CoV infection, while highly diluted anti-sera significantly increased SARS-CoV infection (Wang et al, 2014). In another study certain epitopes against the S protein enhanced infection in rhesus macaques, and was attributed to antibody-dependent disease enhancement (ADE) 176, 177. Therefore, even with the selection of perfect adjuvant and appropriate antigen or antigens for SARS-CoV-2 vaccine development, ADE remains a concern when neutralizing antibodies are elicited at sub-neutralizing doses. Randomized, placebo-controlled clinical trials with existing drugs approved for other diseases is an attractive strategy since it may reduce the costs and time. Furthermore, with the existing pharmaceutical supply chain for formulation and distribution there is the possibility of rapid repositioning of drugs for COVID-19 treatment.

In summation, the COVID-19 Treatment Guidelines Panel recommends against the use of the following drugs for the treatment of COVID-19, combination of HCQ and azithromycin because of potential toxicities. The use of lopinavir/ritonavir or other HIV protease inhibitors because of unfavorable pharmacodynamics and negative clinical trial data. We are waiting for the results regarding the therapeutic and vaccine developments related to blocking ACE2 and S protein interactions and inhibiting RAS.

## Conclusion and Future Directions

SARS-Cov2 appears to have gained increased capacity for transmission over time, infecting millions of people globally and causing significant morbidity and mortality. This is seen particularly among the elderly and those with comorbid conditions, including advanced age, hypertension, diabetes, and obesity. The severity of the pandemic is far beyond expectation and much worse than previous coronavirus epidemics, including SARS and MERS, which had a comparatively faster but limited spread. Efforts to minimize transmission, including closure of businesses and limitation of many activities, have had significant and life-changing impacts, including personal economic hardships and damage to global economies. There is significant debate regarding the best way to restore economies and bring people back to work, without enabling a second wave of infection. It is critical that we identify a means to end the current pandemic and prevent the emergence of a “second” wave of infection. While the clinical and pathological features of COVID-19 are similar to those of SARS, there are several gaps on our current understanding of COVID-19 pathogenesis. First, although ACE2 serves as a receptor for SARS-CoV2, the pathogenic role of ACE2 may be more complex but remains unclear. Second, the SARS-CoV-2 infection also downregulates ACE2 expression in ACE2-expressing cells via shedding ACE2 from tissue and reducing the level of ACE2 in the infected cells. Whether these changes in tissue or blood have any prognostic value for predicting the disease progress remains unclear 178. Third, although clinical trial studies with Ang II inhibitors and angiotensin receptor blockers indicates the critical roles of ACE2 COVID-19 pathogenesis, there remains a pressing need to develop effective therapeutic strategies through clinical trials and studies in animal models. Animal models for dissecting the pathogenic role of ACE2 in COVID-19, and for the evaluation of therapeutic strategies targeting to block ACE2 interaction with the S protein and inhibit the immune activation/cytokine storm for modulating severe COVID-19 are also sorely needed. Finally, current and future preclinical and clinical trials of passive antibodies and vaccine strategies focusing on the interaction of ACE2 with the S protein are urgently needed to inform the development of safe and effective vaccines to mitigate morbidity and mortality of COVID-19 infection. Filling these gaps with multiple approaches and effort will add in our understanding of COVID-19 pathogenesis and allow for the identification of targets for therapeutic intervention and the development of safe and effective neutralizing vaccine.
